# Supplementary material for: Oxidative stress, dysfunctional energy metabolism, and destabilizing neurotransmitters altered the cerebral metabolic profile in a rat model of simulated heliox saturation diving to 4.0 MPa
Source: PLoS One. 2023 Mar 14;18(3):e0282700. doi: 10.1371/journal.pone.0282700 (PMC10013885; doi:10.1371/journal.pone.0282700)

**S1 Figure. Metabolic alterations driven by high pressure exposure of 400msw heliox saturation environment and across different brain regions.** Principal component analysis (PCA) scores plot of PC1/PC2 obtained from ^1^H NMR data and colored according to groups of CONC and HSDC (A, R2X= 0.40, Q2 = -0.02), CONH and HSDH (B, R2X= 0.50, Q2 = 0.16), and CONS and HSDS (C, R2X= 0.43, Q2 = 0.05), ; scaling was done to unit variance; Partial least squares discriminant analysis (PLS-DA) scores plot from ^1^H NMR spectra of extracts from the cortex (A’, R2X= 0.38, Q2 = 0.54), the hippocampus (B’, R2X= 0.37, Q2 = 0.35), and striatum (C’, R2X= 0.39, Q2 = 0.42) from CON and HSD groups; scaling was done to unit variance.


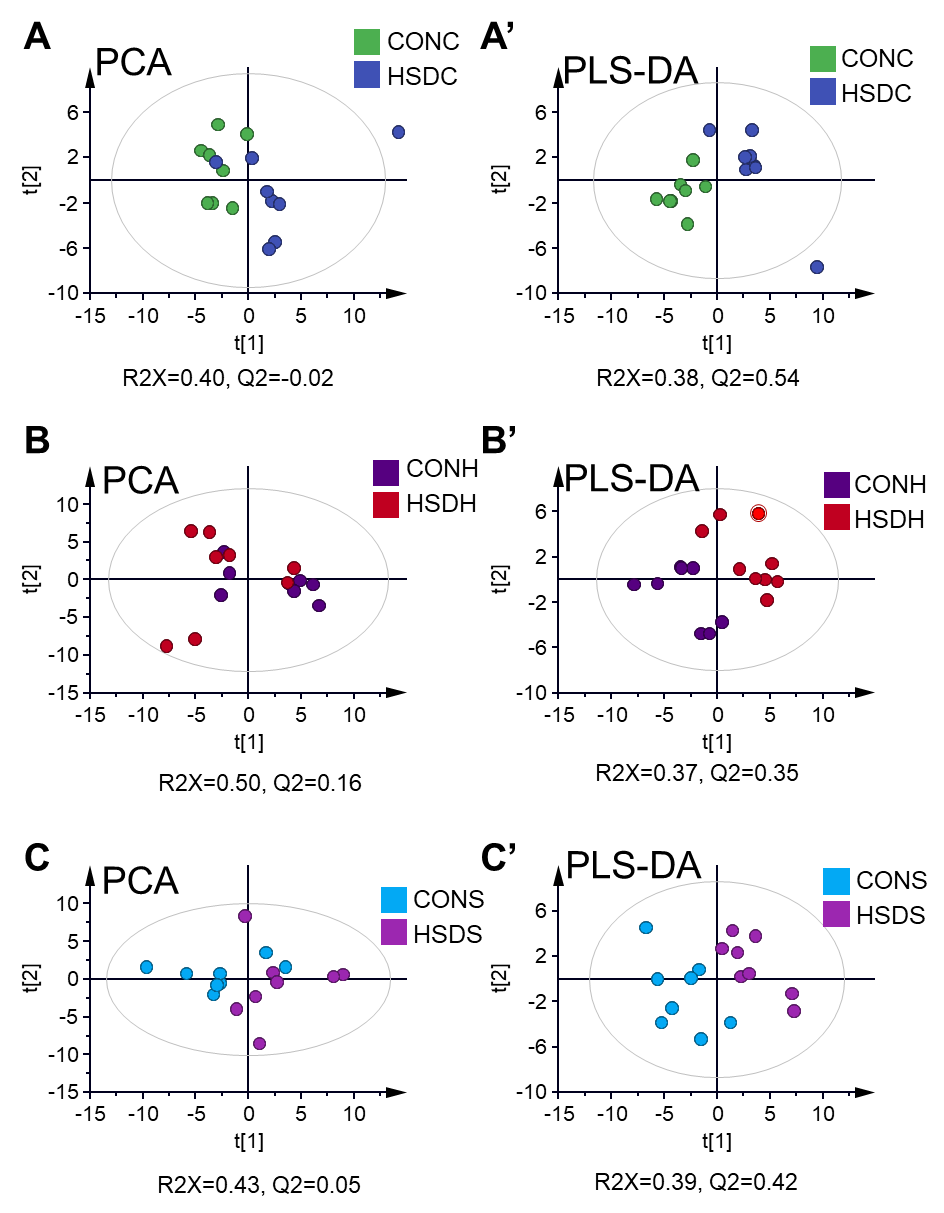

Supplement: S1 Fig — Principal component analysis (PCA) scores plot of PC1/PC2 obtained from 1H NMR data and colored according to groups of CONC and HSDC (A, R2X = 0.40, Q2 = -0.02), CONH and HSDH (B, R2X = 0.50, Q2 = 0.16), and CONS and HSDS (C, R2X = 0.43, Q2 = 0.05),; scaling was done to unit variance; Partial least squares discriminant analysis (PLS-DA) scores plot from 1H NMR spectra of extracts from the cortex (A’, R2X = 0.38, Q2 = 0.54), the hippocampus (B’, R2X = 0.37, Q2 = 0.35), and striatum (C’, R2X = 0.39, Q2 = 0.42) from CON and HSD groups; scaling was done to unit variance. (DOCX) [file pone.0282700.s001.docx]
