# Supplementary material for: Oxidative stress, dysfunctional energy metabolism, and destabilizing neurotransmitters altered the cerebral metabolic profile in a rat model of simulated heliox saturation diving to 4.0 MPa
Source: PLoS One. 2023 Mar 14;18(3):e0282700. doi: 10.1371/journal.pone.0282700 (PMC10013885; doi:10.1371/journal.pone.0282700)
Supplement: S1 Table — (DOCX) [file pone.0282700.s002.docx]

**S1 Table. NMR Resonance assignments of 47 aqueous metabolites in ^1^H NMR spectra of brain samples.**

| Metabolite (abbreviation) | Groups | δ^1^H(ppm) in PBS buffer (pH=7.2) ^#^ |
| --- | --- | --- |
| 2-aminobutyrate (2AB) | α-CH, β-CH_2_, g-CH_3_ | 3.71(t), 1.90(m), 0.97(t) |
| 2-dimethylamine (DMA) | CH_3_ | 2.72(s) |
| 2-hydroxyisobutyrate (2HIB) | 2 X β-CH3 | 1.35(s) |
| 5'-monophosphate (AMP) | ^2^CH, ^8^CH, NH_2_ | 8.59(s), 8.26(s), 6.13(d) |
| Acetate (Ace) | CH3 | 1.90(s) |
| Adenosine triphosphate (ATP) | adenine moity: ^2^CH, ^8^CH, NH2 | 8.50(s), 8.23(s), 6.14(d) |
| Analine (Ala) | α-CH, β-CH_3_ | 3.78(q), 1.47(d) |
| Asparagine (Asn) | α-CH, half β-CH_2_, half β-CH_2_ | 3.99(dd), 2.94(dd), 2.86(dd) |
| Aspartate (Asp) | α-CH, half β-CH_2_, half β-CH_2_ | 3.89(dd), 2.80(dd), 2.68(dd) |
| Ascorbate (Asc) | α-CH, half β-CH_2_, half β-CH_2_ | 3.89(dd), 2.80(dd), 2.68(dd) |
| Carnitine (Car) | CH_3_ | 3.22(s) |
| Choline (Cho) | ^1^CH_2_-OH, ^2^CH_2_-N, N(CH_3_)_3_ | 4.04(m), 3.50(m), 3.19(s) |
| Creatine (Cre) | α-CH_2_, N-CH_3_ | 3.92(s), 3.02(s) |
| Cytidine (Cyt) | cytosine moity: α-CH, β-CH | 7.83(d), 6.05(d) |
| Formate (For) | CH | 8.46(s) |
| Fumarate (FMA) | 2×CH | 6.50(s) |
| Glutamate (Glu) | α-CH, half β-CH_2_, half β-CH_2_, half γ-CH_2_, half γ-CH_2_ | 3.76(t), 2.13(m), 2.05(m), 2.33(m), 2.35(m) |
| Glutamine (Gln) | α-CH, half β-CH_2_,half β-CH2, half γ-CH_2_ , half γ-CH_2_ | 3.77(t), 2.46(m), 2.44(m), 2.14(m), 2.10(m) |
| Glutathione (GSH) | Glutamate moity: α-CH, β-CH_2_, γ-CH_2_. Cystein moity: β-CH2, α-CH. Glycine moity: α-CH2 | 3.79(t), 2.16(m), 2.55(m), 2.98(dd), 4.21(m) , 3.79(m) |
| Glycine (Gly) | α-CH_2_ | 3.55(s) |
| Inosine (Ino) | Hypoxanthine moity: α-CH, α'-CH | 8.23(s), 8.33(s) |
| inosine monophosphate (IMP) | Hypoxanthine moity:α-CH, α'-CH | 8.23(s), 8.57(s) |
| Isoleucine (Ile) | α-CH, β-CH, g-CH_3_, half g-CH_2_, half g-CH_2_, d-CH_3_ | 3.66(d), 2.00(m), 1.24(d), 1.44 (m), 1.00(d), 0.94(t) |
| Lactate (Lac) | α-CH, β-CH3 | 4.10 (q), 1.32(d) |
| Leucine (Leu) | α-CH, half β-CH_2_, half β-CH_2_, g-CH, d-CH_3_, d'-CH_3_ | 3.73(m), 1.73(m), 1.70(m),1.67(m), 0.95(d), 0.94(d) |
| Lysine (Lys) | α-CH, half β-CH_2_, half β-CH_2_, g-CH, d-CH_3_, d'-CH_3_ | 3.00(t), 1.90(m), 1.91(m), 1.50(m), 1.42(m), 1.70(m), 3.75(t) |
| Malate (Mal) | α-CH, β-CH_2_ | 2.66(dd), 2.37(dd) |
| Malonate (Maln) | CH_2_ | 3.14(s) |
| myo-Inositol (MI) | 1CH, 2CH, 3CH, 4CH, 5CH, 6CH | 3.53(dd), 4.06(t), 3.53(dd), 3.62(t), 3.28(t), 3.62(t) |
| N-acetyl-aspartate (NAA) | Acetyl moity: CH_3_ | 2.01(s) |
| Nicotinamide adenine dinucleotide (NAD+) | Nicotinamide moity: α’-CH, α-CH, γ-CH, β-CH | 9.33(s), 8.82(d), 8.17(q), 9.13(d) |
| Nicotinuric acid (Nic) | α’-CH, α-CH, γ-CH, β-CH | 8.93(d), 8.69(dd), 7.59(q), |
| O-Phosphocholine (PCho) | ^1^CH_2_, ^2^CH_2_, N(CH_3_)_3_ | 4.17(m), 3.59(t), 3.21(s) |
| O-phosphoethanolamine (PEA) | N-CH_2_, O-CH_2_ | 3.22(t), 4.01(t) |
| Phenylalanine (Phe) | phenyl moity: α-CH, β-CH, γ-CH, half β-CH_2_, half β-CH_2_, α-CH | 7.33(d), 7.43(t), 7.37(t), 3.98(dd), 3.27(dd), 3.12(dd) |
| Serine (Ser) | α-CH, β-CH, g-CH_3_, g-CH_3_ | 3.98(q), 3.94(q), 3.84(q) |
| sn-glycero-3-phosphocholine (GPC) | glycerol moity: ^1^CH_2_, ^2^CH_2_, N(CH_3_)_3_, half ^1^CH_2_, half ^1^CH_2_, ^2^CH, half ^3^CH_2_, half ^3^CH_2_ | 4.32(m), 3.67(m), 3.22(s), 3.59(dd), 3.68(dd), 3.90(m), 3.87(m), 3.94(m) |
| Succinate (Suc) | CH_3_ | 2.40(s) |
| Taurine (Tau) | ^1^CH_2_, ^2^CH_2_ | 3.40(t), 3.26(t) |
| Threonine (Thr) | α-CH, β-CH, γ-CH_3_ | 3.58(d), 4.26(m), 1.34(d) |
| Tyrosine (Tyr) | phenyl moiety(α-CH, β-CH), α-CH, half β-CH_2_, half β-CH_2_ | 7.19(d), 6.90(d), 3.93(q), 3.05(dd), 3.19(dd) |
| UDP-galactose (UDPGa) | nucleobase uracil moity:α-CH, β-CH | 7.94(d), 5.98(d) |
| Uracil (Ura) | α-CH, β-CH | 7.54(d), 6.80(d) |
| uridine 5'-monophosphate (UMP) | nucleobase uracil moity:α-CH, β-CH | 8.12(d), 5.99(d) |
| Uridine (Uri) | ^1^CH, ^2^CH, ^3^CH, ^4^CH, half CH_2_, half CH_2_, α-CH, β-CH | 5.93(d), 4.34(t), 4.22(t), 4.12(m), 3.90(dd), 3.80(dd), 7.88(d), 5.91(d) |
| Valine (Val) | α-CH, β-CH, g-CH_3_, g-CH_3_ | 3.61(d), 2.27(m), 1.02(d), 0.98(d) |
| γ-aminobutyric acid (GABA) | α-CH_2_, β-CH_2_, γ-CH_2_ | 2.28(t), 1.89(m),3.00(t) |

Note: # s, singlet；d, doublet；t, triplet；q, quartet；m, multiplet.
