# Supplementary material for: Oxidative stress, dysfunctional energy metabolism, and destabilizing neurotransmitters altered the cerebral metabolic profile in a rat model of simulated heliox saturation diving to 4.0 MPa
Source: PLoS One. 2023 Mar 14;18(3):e0282700. doi: 10.1371/journal.pone.0282700 (PMC10013885; doi:10.1371/journal.pone.0282700)
Supplement: S2 Table — (DOCX) [file pone.0282700.s003.docx]

**S2 Table. The buckets mean of** **peak height from discriminative aqueous metabolites in ^1^H NMR spectra acquired on the cerebral, hippocampus, striatum samples of HSD rats comparted to that of CON rats.**

| Metabolites | Cortex | | Hippocampus | | striatum | |
| --- | --- | --- | --- | --- | --- | --- |
|  | CON | HSD | CON | HSD | CON | HSD |
| TP | 1.63±0.43 | 1.42±0.47 | 1.95±0.45 | 1.41±0.44 | 2.33±1 | 1.45±0.38 |
| Ala | 16.67±1.32 | 15.52±1.5 | 20.6±2.2 | 18.39±2.06 | 17.98±2.49 | 16.41±2.18 |
| AMP | 10.98±1.91 | 14.39±1.39 | 7.45±0.45 | 8.17±0.85 | 13.35±4.25 | 13.04±2.29 |
| Asc | 16.86±1.44 | 15.67±1.73 | 17.82±2.01 | 15.49±2 | 11.19±1.83 | 11.69±2.52 |
| Asn | 1.27±0.26 | 1.09±0.07 | 1.45±0.36 | 1.29±0.17 | 1.35±0.27 | 1.52±0.11 |
| Asp | 14.56±1.13 | 13.99±0.97 | 11.46±0.38 | 11.7±0.67 | 11.37±0.72 | 10.53±0.93 |
| Car | 3.13±0.3 | 2.56±0.56 | 2.56±0.11 | 2.62±0.32 | 2.96±0.29 | 2.64±0.36 |
| Cho | 14.98±4.14 | 9.55±2.8 | 13.06±3.29 | 10.45±2.63 | 25.85±4.69 | 16.21±3.39 |
| Cyt | 0.27±0.04 | 0.22±0.05 | 0.33±0.08 | 0.22±0.06 | 0.35±0.03 | 0.22±0.1 |
| DMA | 3.34±0.73 | 4.15±1.71 | 16.16±7.81 | 6.34±2.21 | 16.61±13.26 | 12.07±11.42 |
| FMA | 0.57±0.09 | 0.71±0.2 | 0.45±0.08 | 0.43±0.18 | 0.41±0.25 | 0.41±0.07 |
| GABA | 15.88±1.64 | 14.2±1.41 | 18.03±1.04 | 16.54±0.71 | 18.1±2.58 | 15.57±1.8 |
| Gln | 27.05±2.14 | 26.36±2.22 | 24.17±2.42 | 22.4±2.71 | 26.13±2.45 | 23.5±2.64 |
| Gly | 20.71±1.08 | 23.36±6.21 | 28.67±2.28 | 25.58±2.58 | 23.21±2.89 | 19.93±3.25 |
| GSH | 1.31±0.27 | 0.99±0.1 | 1.52±0.34 | 1.24±0.14 | 1.33±0.28 | 1.59±0.75 |
| Ile | 3.12±0.23 | 3.34±0.36 | 2.82±0.21 | 3±0.74 | 3.78±0.41 | 4.21±0.26 |
| IMP | 3.61±0.52 | 3.2±0.76 | 3.48±0.31 | 2.74±0.5 | 3.48±0.39 | 2.86±0.89 |
| Ino | 1.36±0.3 | 0.71±0.2 | 0.96±0.38 | 0.8±0.31 | 1.02±0.37 | 0.69±0.27 |
| Lac | 192.39±15.68 | 169.73±17.57 | 228.41±24.25 | 197.69±24.25 | 149.03±11.27 | 140.13±34.74 |
| Leu | 5.36±0.34 | 5.51±0.4 | 5.36±0.19 | 5.7±1.12 | 6.96±0.56 | 7.56±0.26 |
| Lys | 3.67±0.15 | 3.83±0.24 | 3.68±0.17 | 3.81±0.53 | 4.79±0.26 | 5.02±0.29 |
| NAA | 213.76±30.27 | 231.42±28.32 | 211.42±17.31 | 195.81±39.11 | 165.63±18.6 | 144.57±27.22 |
| NAD | 0.95±0.06 | 0.89±0.12 | 0.66±0.07 | 0.67±0.1 | 0.76±0.09 | 0.64±0.11 |
| NADP | 0.07±0.02 | 0.09±0.02 | 0.06±0.03 | 0.06±0.02 | 0.21±0.09 | 0.13±0.09 |
| Nic | 0.2±0.03 | 0.27±0.05 | 0.38±0.07 | 0.37±0.08 | 0.59±0.12 | 0.53±0.09 |
| Pcho | 42.47±4.43 | 37.55±5.44 | 45.38±3.29 | 43.48±4.53 | 67.27±6.94 | 58.72±10.9 |
| Phe | 0.26±0.03 | 0.31±0.05 | 0.31±0.04 | 0.29±0.05 | 0.45±0.11 | 0.42±0.08 |
| Suc | 27.63±4.03 | 28.2±4.45 | 27.29±5.24 | 21.42±3.5 | 25.48±5.04 | 21.21±4.07 |
| Tau | 77.55±8.48 | 74.98±6.59 | 79.97±8.04 | 73.45±11.53 | 99.69±13.25 | 78.42±15.03 |
| Thr | 5.67±0.28 | 5.76±0.32 | 5.26±0.32 | 5.74±0.31 | 6.33±0.18 | 6.38±0.43 |
| Tyr | 0.8±0.08 | 0.62±0.15 | 0.67±0.09 | 0.61±0.09 | 0.74±0.2 | 0.55±0.13 |
| Ura | 0.15±0.03 | 0.12±0.04 | 0.11±0.04 | 0.04±0.06 | 0.32±0.08 | 0.22±0.12 |
| Uri | 0.56±0.08 | 0.38±0.07 | 0.41±0.09 | 0.32±0.11 | 0.23±0.15 | 0.19±0.08 |
| Val | 3.86±0.28 | 3.98±0.33 | 3.74±0.17 | 3.89±0.62 | 4.63±0.41 | 5.05±0.33 |
