# Supplementary material for: Oxidative stress, dysfunctional energy metabolism, and destabilizing neurotransmitters altered the cerebral metabolic profile in a rat model of simulated heliox saturation diving to 4.0 MPa
Source: PLoS One. 2023 Mar 14;18(3):e0282700. doi: 10.1371/journal.pone.0282700 (PMC10013885; doi:10.1371/journal.pone.0282700)
Supplement: S3 Table — (DOCX) [file pone.0282700.s004.docx]

**S3 Table. The limit of detection of assay kits used in the present work.**

| Company | Product name | Limit of detection |
| --- | --- | --- |
| Abcam | ab83355 ATP Assay Kit | 1 µM |
| Abcam | ab235937 Cholinesterase Activity Assay | 1 mU/ml |
| Abcam | ab102526 Lactate Dehydrogenase Assay | 1 mU/ml |
| RD | Universal Dopamine ELISA Kit | 18.75 pg/mL |
| Abnova | KA3768 Epinephrine/Norepinephrine ELISA Kit | Adrenaline: 0.25 ng/mL |
| Abnova | KA3768 Epinephrine/Norepinephrine ELISA Kit | Noradrenaline: 0.1 ng/mL |
| BioSource | Rat 5-Hydroxytryptamine ELISA Kit | 1.0 ng/mL |
| Santa Cruz | Rat Gamma-aminobutyric acid (GABA) ELISA Kit | 0.1μmol/L |
| cayman | Rat Super Oxidase Dimutase (SOD) ELISA Kit | 1.0 U/mL |
| cayman | Rat malondialchehyche (MDA) ELISA Kit | 0.1 nmol/mL |
| cayman | Rat Glutathione peroxidase (GSH-Px) ELISA Kit | 1.0 U/mL |
