# Supplementary material for: Oxidative stress, dysfunctional energy metabolism, and destabilizing neurotransmitters altered the cerebral metabolic profile in a rat model of simulated heliox saturation diving to 4.0 MPa
Source: PLoS One. 2023 Mar 14;18(3):e0282700. doi: 10.1371/journal.pone.0282700 (PMC10013885; doi:10.1371/journal.pone.0282700)
Supplement: S4 Table — (DOCX) [file pone.0282700.s005.docx]

**S4 Table. The peak height raw data of cortex samples.**

| Metabolites | Cortex | | | | | | | | | | | | | | | |
| --- | --- | --- | --- | --- | --- | --- | --- | --- | --- | --- | --- | --- | --- | --- | --- | --- |
|  | CON1 | CON2 | CON3 | CON4 | CON5 | CON6 | CON7 | CON8 | HSD1 | HSD2 | HSD3 | HSD4 | HSD5 | HSD6 | HSD7 | HSD8 |
| 2HIB | 8.52 | 8.43 | 8.49 | 9.77 | 8.91 | 9.51 | 8.41 | 8.37 | 8.19 | 9.57 | 8.91 | 7.95 | 9.18 | 9.39 | 9.46 | 9.96 |
| Ala | 16.20 | 18.65 | 15.01 | 16.84 | 16.00 | 15.60 | 18.57 | 16.47 | 16.40 | 15.18 | 15.75 | 16.58 | 15.45 | 12.03 | 16.35 | 16.42 |
| AMP1 | 15.31 | 15.82 | 16.28 | 13.43 | 9.04 | 14.75 | 12.20 | 16.94 | 15.49 | 16.18 | 11.36 | 12.39 | 14.50 | 16.56 | 18.61 | 11.19 |
| AMP2 | 12.02 | 11.72 | 11.67 | 8.14 | 11.85 | 11.76 | 7.78 | 12.90 | 13.00 | 14.02 | 12.36 | 14.86 | 14.77 | 14.82 | 16.98 | 14.27 |
| AMP3 | 5.18 | 5.81 | 6.52 | 5.22 | 6.88 | 6.14 | 5.02 | 7.54 | 8.18 | 6.14 | 7.08 | 9.04 | 8.24 | 8.05 | 6.34 | 8.45 |
| AMP4 | 6.30 | 6.20 | 6.54 | 6.14 | 6.28 | 6.22 | 5.85 | 6.34 | 7.15 | 6.40 | 6.35 | 6.98 | 5.80 | 6.09 | 7.02 | 5.20 |
| AMP5 | 6.30 | 6.20 | 6.54 | 6.14 | 6.28 | 6.22 | 5.85 | 6.34 | 7.15 | 6.41 | 6.36 | 6.99 | 5.81 | 6.09 | 7.02 | 5.20 |
| AMP6 | 8.20 | 7.24 | 7.32 | 7.48 | 7.48 | 8.00 | 7.67 | 7.28 | 8.33 | 7.42 | 7.65 | 8.55 | 8.33 | 7.36 | 7.91 | 8.43 |
| Asc | 17.08 | 17.92 | 18.17 | 14.69 | 16.42 | 14.75 | 17.94 | 17.89 | 16.58 | 16.31 | 15.29 | 17.20 | 16.19 | 17.56 | 13.22 | 12.97 |
| Asn | 1.84 | 1.16 | 1.27 | 1.39 | 1.23 | 0.99 | 1.19 | 1.10 | 1.23 | 1.11 | 1.03 | 1.10 | 1.05 | 1.12 | 1.06 | 1.00 |
| Asp1 | 14.34 | 14.86 | 16.88 | 13.25 | 14.78 | 14.94 | 13.67 | 13.76 | 13.74 | 15.80 | 14.25 | 14.39 | 12.44 | 13.70 | 13.32 | 14.30 |
| Asp2 | 12.29 | 10.36 | 13.01 | 9.60 | 8.28 | 12.66 | 7.50 | 9.83 | 10.90 | 8.89 | 9.37 | 7.88 | 6.82 | 8.47 | 9.61 | 10.32 |
| Asp3 | 12.34 | 12.17 | 15.05 | 10.82 | 11.82 | 13.15 | 11.16 | 10.43 | 11.35 | 13.32 | 10.85 | 11.17 | 9.31 | 11.16 | 10.05 | 7.70 |
| ATP | 1.25 | 1.63 | 1.24 | 1.55 | 2.50 | 1.28 | 1.91 | 1.68 | 1.17 | 1.02 | 1.72 | 2.34 | 1.71 | 0.97 | 1.14 | 1.32 |
| Car | 3.50 | 3.19 | 2.97 | 3.49 | 2.66 | 3.01 | 3.33 | 2.87 | 2.97 | 2.40 | 2.11 | 3.62 | 2.32 | 2.51 | 2.76 | 1.82 |
| Cho | 17.56 | 20.15 | 13.89 | 17.07 | 14.33 | 9.30 | 18.58 | 9.01 | 9.42 | 7.81 | 8.38 | 11.87 | 8.44 | 5.29 | 10.69 | 14.49 |
| Cre | 219.15 | 208.75 | 225.96 | 194.43 | 220.24 | 222.80 | 215.10 | 210.34 | 212.10 | 215.06 | 208.43 | 203.62 | 185.52 | 217.05 | 222.42 | 144.83 |
| Cyt | 0.25 | 0.33 | 0.32 | 0.30 | 0.24 | 0.24 | 0.30 | 0.20 | 0.16 | 0.20 | 0.21 | 0.18 | 0.18 | 0.23 | 0.30 | 0.30 |
| DMA | 3.35 | 3.28 | 2.44 | 3.67 | 2.60 | 4.83 | 3.19 | 3.37 | 3.97 | 5.25 | 2.74 | 2.34 | 3.11 | 4.47 | 7.67 | 3.61 |
| FMA | 0.63 | 0.50 | 0.63 | 0.61 | 0.54 | 0.70 | 0.49 | 0.45 | 0.82 | 0.27 | 0.76 | 0.74 | 0.82 | 0.64 | 0.93 | 0.73 |
| For | 1.04 | 0.82 | 0.75 | 0.82 | 0.58 | 1.12 | 0.63 | 1.22 | 0.92 | 1.06 | 0.86 | 0.69 | 0.85 | 0.78 | 3.00 | 1.87 |
| GABA1 | 18.25 | 18.57 | 21.31 | 17.07 | 17.41 | 19.10 | 18.90 | 19.17 | 19.73 | 15.71 | 20.07 | 22.10 | 19.59 | 19.03 | 18.16 | 23.33 |
| GABA2 | 14.13 | 18.21 | 15.64 | 14.55 | 15.82 | 13.93 | 17.27 | 17.52 | 14.20 | 14.17 | 14.62 | 16.81 | 13.02 | 13.47 | 15.17 | 12.18 |
| GABA3 | 16.04 | 19.95 | 16.50 | 16.13 | 17.24 | 15.85 | 18.83 | 19.41 | 16.29 | 15.12 | 16.67 | 19.34 | 15.46 | 13.96 | 16.91 | 16.26 |
| Gln1 | 28.69 | 33.22 | 33.38 | 29.35 | 31.76 | 29.33 | 33.33 | 35.88 | 28.39 | 34.28 | 27.98 | 31.55 | 26.69 | 35.34 | 29.10 | 18.58 |
| Gln2 | 27.75 | 27.40 | 23.32 | 24.80 | 27.26 | 29.52 | 26.81 | 29.53 | 27.69 | 27.78 | 28.51 | 28.92 | 26.38 | 24.05 | 24.35 | 23.15 |
| Glu1 | 34.43 | 32.30 | 45.72 | 27.80 | 35.15 | 36.46 | 38.87 | 32.21 | 36.85 | 36.15 | 33.49 | 31.93 | 30.31 | 43.17 | 34.67 | 39.29 |
| Glu2 | 53.59 | 52.14 | 48.18 | 47.77 | 52.84 | 58.20 | 51.31 | 53.49 | 58.48 | 51.56 | 54.79 | 55.69 | 53.74 | 45.48 | 55.45 | 54.77 |
| Glu3 | 53.92 | 52.41 | 48.45 | 48.00 | 53.12 | 58.51 | 51.60 | 53.78 | 58.80 | 51.82 | 55.08 | 55.98 | 54.01 | 45.74 | 55.77 | 55.03 |
| Gly | 20.29 | 22.25 | 19.08 | 19.79 | 20.04 | 21.55 | 21.01 | 21.66 | 21.97 | 19.78 | 27.29 | 36.29 | 20.02 | 17.64 | 25.45 | 18.43 |
| GPC | 43.22 | 45.36 | 48.37 | 36.32 | 49.68 | 46.08 | 41.10 | 55.16 | 45.50 | 56.88 | 50.27 | 58.49 | 56.04 | 41.48 | 40.75 | 30.15 |
| GSH1 | 1.96 | 1.33 | 1.34 | 1.43 | 1.56 | 1.17 | 1.20 | 1.00 | 1.26 | 1.01 | 1.20 | 1.05 | 1.48 | 1.09 | 0.94 | 1.41 |
| GSH2 | 3.89 | 4.33 | 4.38 | 3.88 | 3.82 | 3.95 | 4.33 | 4.57 | 4.12 | 4.46 | 3.74 | 4.65 | 3.50 | 4.65 | 4.15 | 3.04 |
| GSH3 | 5.37 | 4.69 | 4.15 | 4.36 | 4.63 | 4.34 | 4.17 | 4.22 | 4.59 | 4.04 | 3.92 | 4.61 | 4.53 | 3.72 | 3.97 | 3.49 |
| GSH4 | 1.78 | 1.30 | 1.33 | 1.49 | 1.41 | 1.00 | 1.20 | 0.95 | 1.22 | 0.97 | 1.03 | 0.99 | 0.96 | 0.92 | 0.94 | 0.88 |
| Ile | 2.96 | 3.27 | 2.79 | 3.30 | 3.04 | 3.18 | 3.48 | 2.91 | 3.26 | 3.38 | 3.41 | 3.09 | 3.03 | 3.07 | 3.35 | 4.16 |
| IMP1 | 1.57 | 0.88 | 0.96 | 0.90 | 0.81 | 2.69 | 1.40 | 1.71 | 1.62 | 1.59 | 1.39 | 0.75 | 2.80 | 2.39 | 2.46 | 0.70 |
| IMP2 | 3.67 | 3.62 | 3.74 | 3.70 | 3.81 | 3.45 | 4.39 | 2.51 | 3.20 | 2.74 | 3.37 | 3.55 | 3.19 | 1.84 | 4.57 | 3.12 |
| Ino | 1.49 | 1.63 | 1.59 | 1.59 | 1.19 | 1.07 | 1.50 | 0.81 | 0.70 | 0.99 | 0.65 | 0.78 | 0.63 | 0.62 | 0.96 | 0.39 |
| Lac1 | 36.44 | 37.24 | 37.92 | 37.60 | 33.78 | 44.39 | 41.60 | 40.87 | 36.09 | 33.40 | 32.85 | 28.43 | 40.76 | 33.73 | 44.47 | 32.23 |
| Lac2 | 186.34 | 199.71 | 159.18 | 192.66 | 188.95 | 210.37 | 196.12 | 205.80 | 183.11 | 167.34 | 177.68 | 167.43 | 179.62 | 132.65 | 188.80 | 161.25 |
| Leu | 5.09 | 5.66 | 5.14 | 5.59 | 5.10 | 5.24 | 5.98 | 5.10 | 5.37 | 5.77 | 5.21 | 5.33 | 5.17 | 5.58 | 5.28 | 6.38 |
| Lys | 3.64 | 3.77 | 3.59 | 3.85 | 3.75 | 3.64 | 3.78 | 3.37 | 3.86 | 3.77 | 3.69 | 4.01 | 3.81 | 3.63 | 3.57 | 4.31 |
| Mal | 6.42 | 7.41 | 10.21 | 6.59 | 7.99 | 6.93 | 7.52 | 6.05 | 6.90 | 8.67 | 7.12 | 7.97 | 6.50 | 7.87 | 5.81 | 5.04 |
| MI | 35.99 | 35.39 | 23.25 | 31.74 | 29.99 | 34.62 | 31.19 | 36.14 | 35.00 | 28.27 | 32.33 | 32.03 | 33.35 | 24.48 | 30.86 | 32.50 |
| MI1 | 52.97 | 68.36 | 69.63 | 52.22 | 57.33 | 50.62 | 68.61 | 73.51 | 50.53 | 64.55 | 49.52 | 52.58 | 49.95 | 69.43 | 51.63 | 39.99 |
| NAA1 | 20.81 | 20.65 | 23.01 | 18.94 | 21.73 | 21.58 | 20.64 | 20.11 | 20.81 | 20.40 | 20.04 | 21.00 | 20.42 | 22.18 | 21.07 | 21.36 |
| NAA2 | 245.27 | 200.78 | 230.93 | 197.95 | 216.01 | 261.31 | 176.05 | 181.81 | 251.64 | 164.26 | 242.76 | 232.38 | 233.00 | 234.72 | 238.88 | 253.72 |
| NAA3 | 34.56 | 32.79 | 32.87 | 29.65 | 33.05 | 36.00 | 30.66 | 30.29 | 33.37 | 32.54 | 31.83 | 32.91 | 29.91 | 27.62 | 31.57 | 28.54 |
| NAA4 | 32.36 | 32.85 | 35.74 | 29.49 | 33.91 | 33.47 | 32.45 | 32.12 | 31.94 | 32.14 | 31.03 | 33.31 | 30.04 | 33.92 | 31.86 | 24.96 |
| NAD1 | 1.64 | 1.71 | 1.51 | 1.52 | 1.69 | 1.76 | 1.77 | 1.48 | 1.50 | 1.72 | 1.64 | 1.32 | 1.57 | 1.61 | 1.65 | 1.27 |
| NAD2 | 0.96 | 1.00 | 0.93 | 0.88 | 0.97 | 1.03 | 1.05 | 0.96 | 0.87 | 1.05 | 0.94 | 0.75 | 0.87 | 1.00 | 0.97 | 0.75 |
| NAD3 | 0.90 | 0.97 | 0.94 | 0.86 | 0.94 | 1.01 | 1.03 | 0.94 | 0.86 | 1.02 | 0.94 | 0.74 | 0.88 | 0.99 | 1.02 | 0.71 |
| NADP1 | 0.11 | 0.14 | 0.11 | 0.10 | 0.15 | 0.11 | 0.16 | 0.11 | 0.13 | 0.14 | 0.15 | 0.12 | 0.15 | 0.16 | 0.15 | 0.12 |
| NADP2 | 0.08 | 0.07 | 0.06 | 0.10 | 0.07 | 0.10 | 0.09 | 0.09 | 0.08 | 0.10 | 0.08 | 0.07 | 0.09 | 0.09 | 0.12 | 0.09 |
| NADP3 | 0.08 | 0.06 | 0.06 | 0.05 | 0.03 | 0.08 | 0.05 | 0.10 | 0.10 | 0.08 | 0.07 | 0.06 | 0.06 | 0.09 | 0.11 | 0.13 |
| Nic1 | 0.28 | 0.20 | 0.33 | 0.25 | 0.28 | 0.28 | 0.34 | 0.22 | 0.45 | 0.26 | 0.32 | 0.41 | 0.33 | 0.27 | 0.36 | 0.39 |
| Nic2 | 0.20 | 0.19 | 0.25 | 0.19 | 0.20 | 0.21 | 0.23 | 0.16 | 0.30 | 0.20 | 0.26 | 0.32 | 0.24 | 0.24 | 0.28 | 0.33 |
| Nic3 | 0.21 | 0.17 | 0.27 | 0.22 | 0.21 | 0.19 | 0.21 | 0.16 | 0.29 | 0.19 | 0.22 | 0.33 | 0.19 | 0.20 | 0.22 | 0.32 |
| Pcho | 44.82 | 47.59 | 40.91 | 37.98 | 37.92 | 39.10 | 41.87 | 49.58 | 42.70 | 41.79 | 38.86 | 42.73 | 36.20 | 38.32 | 32.64 | 27.17 |
| PEA | 9.76 | 12.00 | 11.43 | 9.35 | 10.20 | 8.92 | 11.32 | 11.90 | 9.66 | 11.20 | 8.92 | 9.92 | 8.47 | 11.28 | 9.63 | 7.16 |
| Phe1 | 0.43 | 0.48 | 0.52 | 0.48 | 0.49 | 0.56 | 0.53 | 0.50 | 0.53 | 0.47 | 0.49 | 0.46 | 0.40 | 0.51 | 0.53 | 0.49 |
| Phe2 | 0.23 | 0.26 | 0.28 | 0.23 | 0.28 | 0.26 | 0.24 | 0.30 | 0.33 | 0.26 | 0.29 | 0.31 | 0.29 | 0.34 | 0.27 | 0.43 |
| Phe3 | 0.48 | 0.54 | 0.52 | 0.52 | 0.53 | 0.60 | 0.58 | 0.49 | 0.63 | 0.48 | 0.54 | 0.59 | 0.49 | 0.57 | 0.55 | 0.68 |
| Ser1 | 10.31 | 9.98 | 9.57 | 9.42 | 9.55 | 10.45 | 9.74 | 10.52 | 9.68 | 10.64 | 9.23 | 9.91 | 9.79 | 9.73 | 9.33 | 8.73 |
| Ser2 | 7.40 | 7.60 | 7.36 | 7.34 | 6.94 | 7.86 | 7.07 | 7.61 | 7.47 | 7.64 | 7.37 | 7.06 | 6.89 | 6.94 | 7.51 | 7.49 |
| Ser3 | 7.90 | 8.06 | 7.71 | 7.72 | 7.88 | 8.12 | 7.81 | 8.13 | 7.72 | 7.93 | 7.66 | 7.89 | 7.73 | 7.69 | 8.17 | 6.77 |
| Suc | 25.92 | 31.43 | 18.82 | 29.19 | 31.57 | 28.21 | 28.33 | 27.54 | 27.72 | 25.38 | 29.53 | 28.68 | 31.37 | 19.98 | 35.29 | 27.63 |
| Tau1 | 95.29 | 76.12 | 67.50 | 69.18 | 76.47 | 80.51 | 76.01 | 79.32 | 82.91 | 71.73 | 77.10 | 78.10 | 74.76 | 67.54 | 82.72 | 64.94 |
| Tau2 | 104.12 | 87.19 | 73.42 | 78.36 | 82.24 | 89.75 | 84.21 | 91.42 | 92.10 | 79.07 | 85.40 | 86.26 | 82.61 | 74.29 | 90.21 | 71.15 |
| Thr1 | 5.57 | 5.33 | 5.41 | 5.88 | 5.56 | 6.19 | 5.65 | 5.81 | 5.84 | 6.28 | 5.75 | 5.70 | 5.54 | 6.08 | 5.27 | 5.59 |
| Thr2 | 7.54 | 7.46 | 6.98 | 8.30 | 7.29 | 8.56 | 7.65 | 7.93 | 7.75 | 7.89 | 7.73 | 7.51 | 6.33 | 6.68 | 7.06 | 6.72 |
| Tyr1 | 0.80 | 0.84 | 0.79 | 0.85 | 0.76 | 0.69 | 0.95 | 0.73 | 0.51 | 0.88 | 0.46 | 0.48 | 0.57 | 0.73 | 0.78 | 0.58 |
| Tyr2 | 0.82 | 0.97 | 0.84 | 0.87 | 1.00 | 0.73 | 0.99 | 0.88 | 0.65 | 0.94 | 0.68 | 0.68 | 0.63 | 0.77 | 0.77 | 0.75 |
| UDPGa | 1.60 | 1.45 | 1.66 | 1.30 | 1.39 | 1.63 | 1.39 | 1.65 | 1.72 | 1.67 | 1.57 | 1.71 | 1.34 | 1.77 | 1.66 | 0.99 |
| undermine | 3.22 | 2.91 | 2.61 | 2.27 | 2.22 | 4.41 | 2.83 | 3.79 | 2.95 | 3.93 | 2.79 | 2.22 | 4.20 | 4.71 | 3.14 | 1.88 |
| Ura1 | 0.12 | 0.19 | 0.16 | 0.15 | 0.13 | 0.13 | 0.21 | 0.11 | 0.08 | 0.12 | 0.11 | 0.10 | 0.11 | 0.10 | 0.18 | 0.21 |
| Ura2 | 0.14 | 0.19 | 0.26 | 0.16 | 0.19 | 0.15 | 0.22 | 0.13 | 0.08 | 0.17 | 0.16 | 0.09 | 0.24 | 0.15 | 0.18 | 0.07 |
| Uri | 0.53 | 0.68 | 0.63 | 0.54 | 0.58 | 0.50 | 0.62 | 0.42 | 0.33 | 0.43 | 0.41 | 0.44 | 0.43 | 0.34 | 0.45 | 0.25 |
| Val1 | 3.65 | 4.11 | 3.58 | 3.96 | 3.61 | 3.76 | 4.37 | 3.83 | 3.88 | 4.07 | 4.06 | 3.71 | 3.41 | 4.00 | 4.14 | 4.54 |
| Val2 | 3.93 | 4.27 | 3.69 | 4.24 | 3.80 | 3.99 | 4.53 | 3.90 | 4.17 | 4.21 | 4.19 | 3.97 | 3.74 | 4.10 | 4.36 | 4.84 |

* There are two or more peaks of some metabolites in the NMR spectra, and then those peaks were named as the abbreviate name added with a number.
