# Supplementary material for: Oxidative stress, dysfunctional energy metabolism, and destabilizing neurotransmitters altered the cerebral metabolic profile in a rat model of simulated heliox saturation diving to 4.0 MPa
Source: PLoS One. 2023 Mar 14;18(3):e0282700. doi: 10.1371/journal.pone.0282700 (PMC10013885; doi:10.1371/journal.pone.0282700)
Supplement: S5 Table — (DOCX) [file pone.0282700.s006.docx]

**S5 Table. The peak height raw data of Hippocampus samples.**

| Metabolites | Hippocampus | | | | | | | | | | | | | | | |
| --- | --- | --- | --- | --- | --- | --- | --- | --- | --- | --- | --- | --- | --- | --- | --- | --- |
|  | CONH1 | CONH2 | CONH3 | CONH4 | CONH5 | CONH6 | CONH7 | CONH8 | HSDH1 | HSDH2 | HSDH3 | HSDH4 | HSDH5 | HSDH6 | HSDH7 | HSDH8 |
| 2HIB | 8.05 | 7.96 | 10.29 | 12.70 | 11.49 | 9.25 | 10.21 | 10.79 | 8.43 | 10.03 | 10.14 | 11.45 | 7.68 | 8.81 | 7.94 | 8.08 |
| Ala | 22.90 | 18.18 | 20.38 | 17.84 | 23.96 | 18.92 | 21.29 | 21.37 | 16.42 | 18.72 | 18.37 | 21.89 | 20.14 | 18.56 | 17.86 | 15.19 |
| AMP1 | 17.11 | 11.47 | 20.02 | 9.16 | 18.50 | 12.74 | 21.50 | 18.32 | 16.14 | 11.79 | 14.39 | 22.55 | 17.21 | 16.53 | 13.98 | 12.90 |
| AMP2 | 12.09 | 11.86 | 14.29 | 10.43 | 14.15 | 12.04 | 15.89 | 13.72 | 16.42 | 8.59 | 14.17 | 16.41 | 12.80 | 14.26 | 14.09 | 13.86 |
| AMP3 | 6.93 | 9.21 | 7.39 | 8.04 | 8.24 | 10.15 | 7.82 | 8.03 | 10.63 | 6.58 | 7.87 | 8.81 | 7.17 | 10.85 | 9.91 | 10.91 |
| AMP4 | 6.02 | 5.28 | 7.04 | 5.44 | 6.34 | 5.61 | 7.20 | 6.54 | 6.05 | 5.15 | 6.29 | 7.78 | 6.60 | 6.52 | 6.22 | 5.36 |
| AMP5 | 6.02 | 5.28 | 7.04 | 5.44 | 6.34 | 5.61 | 7.20 | 6.54 | 6.05 | 5.16 | 6.29 | 7.79 | 6.61 | 6.52 | 6.22 | 5.36 |
| AMP6 | 6.87 | 7.48 | 7.88 | 6.88 | 7.19 | 7.91 | 8.01 | 7.38 | 8.62 | 6.67 | 7.45 | 9.10 | 7.84 | 8.72 | 7.95 | 9.02 |
| Asc | 18.03 | 18.25 | 17.24 | 15.06 | 18.21 | 20.95 | 19.64 | 15.21 | 14.72 | 14.09 | 14.03 | 16.41 | 14.95 | 17.98 | 18.67 | 13.09 |
| Asn | 2.18 | 1.37 | 1.30 | 1.37 | 1.15 | 1.37 | 1.75 | 1.08 | 1.27 | 1.63 | 1.38 | 1.06 | 1.19 | 1.27 | 1.37 | 1.17 |
| Asp1 | 12.23 | 11.45 | 11.08 | 11.53 | 11.34 | 11.72 | 11.07 | 11.31 | 11.36 | 10.95 | 11.01 | 11.42 | 12.15 | 11.55 | 12.80 | 12.37 |
| Asp2 | 8.91 | 7.23 | 7.92 | 7.89 | 8.04 | 7.77 | 8.24 | 8.08 | 7.24 | 8.38 | 8.17 | 8.45 | 8.82 | 7.60 | 8.67 | 7.32 |
| Asp3 | 8.66 | 8.05 | 7.57 | 7.99 | 8.12 | 8.80 | 8.14 | 7.88 | 8.03 | 9.00 | 8.50 | 7.70 | 8.06 | 8.18 | 9.36 | 8.52 |
| ATP | 2.22 | 2.45 | 1.40 | 2.58 | 1.66 | 1.87 | 1.43 | 2.01 | 1.45 | 1.80 | 0.81 | 0.88 | 1.92 | 1.12 | 1.90 | 1.36 |
| Car | 2.68 | 2.72 | 2.58 | 2.42 | 2.43 | 2.55 | 2.58 | 2.49 | 2.87 | 2.81 | 2.02 | 2.36 | 2.68 | 2.65 | 2.51 | 3.05 |
| Cho | 20.43 | 15.10 | 10.92 | 10.91 | 12.37 | 11.25 | 11.19 | 12.29 | 8.27 | 13.36 | 10.77 | 9.52 | 15.33 | 8.10 | 8.29 | 9.96 |
| Cre | 235.17 | 207.34 | 228.24 | 204.72 | 257.18 | 215.78 | 239.86 | 232.70 | 226.55 | 186.69 | 184.74 | 232.95 | 233.55 | 224.58 | 216.88 | 204.86 |
| Cyt | 0.39 | 0.45 | 0.32 | 0.35 | 0.30 | 0.22 | 0.24 | 0.31 | 0.22 | 0.25 | 0.21 | 0.18 | 0.32 | 0.12 | 0.24 | 0.26 |
| DMA | 11.77 | 3.24 | 23.03 | 23.17 | 23.79 | 7.96 | 20.53 | 15.76 | 6.14 | 3.64 | 6.67 | 10.26 | 7.02 | 6.48 | 7.32 | 3.20 |
| FMA | 0.35 | 0.53 | 0.46 | 0.42 | 0.43 | 0.41 | 0.60 | 0.39 | 0.76 | 0.15 | 0.46 | 0.54 | 0.33 | 0.42 | 0.31 | 0.49 |
| For | 2.06 | 1.95 | 2.23 | 3.87 | 3.29 | 1.34 | 2.06 | 3.30 | 2.35 | 1.60 | 2.98 | 2.38 | 2.53 | 1.66 | 2.08 | 2.03 |
| GABA1 | 24.99 | 27.16 | 21.71 | 23.69 | 19.30 | 27.82 | 21.74 | 21.46 | 25.70 | 19.90 | 18.52 | 21.04 | 22.63 | 24.51 | 23.58 | 26.12 |
| GABA2 | 19.99 | 18.32 | 16.79 | 16.98 | 18.16 | 18.55 | 17.25 | 18.22 | 17.22 | 16.23 | 15.45 | 15.78 | 17.15 | 17.38 | 16.81 | 16.28 |
| GABA3 | 22.75 | 19.64 | 19.17 | 18.25 | 20.08 | 19.26 | 19.58 | 20.45 | 18.27 | 17.26 | 17.04 | 18.13 | 19.93 | 18.85 | 18.27 | 16.61 |
| Gln | 27.00 | 31.82 | 27.08 | 33.60 | 29.52 | 33.26 | 28.07 | 29.12 | 35.44 | 26.64 | 26.29 | 27.99 | 29.32 | 32.06 | 33.89 | 34.30 |
| Gln2 | 26.18 | 20.72 | 25.62 | 20.96 | 23.37 | 23.48 | 26.97 | 26.10 | 22.01 | 19.01 | 21.24 | 27.35 | 24.94 | 21.21 | 23.32 | 20.09 |
| Glu | 36.33 | 36.11 | 35.70 | 29.87 | 35.69 | 38.29 | 36.31 | 36.25 | 34.08 | 25.54 | 30.88 | 37.83 | 36.29 | 36.42 | 34.81 | 36.04 |
| Glu2 | 50.17 | 40.49 | 49.53 | 39.29 | 51.06 | 44.57 | 51.13 | 49.56 | 41.80 | 37.78 | 41.87 | 51.89 | 49.03 | 44.65 | 42.83 | 43.67 |
| Glu3 | 50.42 | 40.72 | 49.80 | 39.50 | 51.38 | 44.82 | 51.40 | 49.82 | 42.05 | 37.96 | 42.04 | 52.18 | 49.34 | 44.91 | 43.05 | 43.90 |
| Gly | 30.54 | 27.90 | 25.48 | 27.08 | 32.08 | 26.46 | 29.88 | 29.95 | 24.25 | 20.01 | 27.03 | 27.00 | 27.61 | 27.11 | 27.06 | 24.58 |
| GPC | 38.62 | 39.40 | 53.30 | 42.46 | 45.80 | 43.35 | 49.65 | 42.60 | 40.61 | 39.86 | 32.57 | 38.58 | 37.49 | 42.69 | 48.36 | 46.18 |
| GSH1 | 2.03 | 1.56 | 1.30 | 1.31 | 1.18 | 1.76 | 1.87 | 1.12 | 1.21 | 1.31 | 1.13 | 1.09 | 1.11 | 1.40 | 1.48 | 1.20 |
| GSH2 | 3.91 | 4.51 | 4.18 | 4.46 | 4.03 | 4.33 | 4.12 | 4.12 | 5.21 | 4.65 | 4.30 | 4.36 | 4.28 | 4.54 | 4.72 | 4.96 |
| GSH3 | 5.64 | 4.56 | 4.83 | 4.08 | 4.86 | 5.07 | 5.57 | 4.31 | 4.04 | 4.34 | 4.28 | 4.66 | 4.60 | 4.24 | 4.59 | 3.62 |
| GSH4 | 1.77 | 1.62 | 1.17 | 1.31 | 1.20 | 2.16 | 1.49 | 1.01 | 1.00 | 1.47 | 1.25 | 1.07 | 1.10 | 1.44 | 1.51 | 0.79 |
| Ile | 3.06 | 2.51 | 2.86 | 2.90 | 2.91 | 2.50 | 2.87 | 2.98 | 2.50 | 4.10 | 4.27 | 2.56 | 2.59 | 2.72 | 2.71 | 2.54 |
| IMP1 | 1.39 | 1.80 | 1.70 | 1.95 | 1.69 | 3.24 | 2.16 | 1.65 | 3.75 | 1.55 | 0.87 | 2.17 | 1.97 | 2.42 | 2.57 | 2.84 |
| IMP2 | 3.93 | 3.98 | 3.37 | 3.26 | 3.37 | 3.48 | 3.35 | 3.12 | 2.64 | 2.12 | 1.97 | 3.26 | 3.11 | 2.68 | 2.76 | 3.36 |
| Ino | 1.76 | 0.87 | 0.89 | 0.76 | 1.06 | 0.44 | 0.80 | 1.07 | 0.38 | 1.10 | 0.87 | 0.81 | 1.33 | 0.45 | 0.71 | 0.73 |
| Lac1 | 56.94 | 57.53 | 48.42 | 49.69 | 57.52 | 51.95 | 49.95 | 53.53 | 48.93 | 40.88 | 45.41 | 48.88 | 50.77 | 46.18 | 48.64 | 49.11 |
| Lac2 | 261.96 | 222.15 | 221.55 | 205.67 | 254.66 | 190.96 | 224.99 | 245.34 | 179.98 | 188.89 | 200.72 | 225.12 | 235.96 | 193.12 | 198.47 | 159.24 |
| Leu | 5.52 | 5.56 | 5.22 | 5.60 | 5.39 | 5.05 | 5.27 | 5.30 | 5.21 | 7.36 | 7.57 | 4.59 | 4.91 | 5.57 | 5.13 | 5.25 |
| Lys | 3.61 | 3.69 | 3.61 | 3.98 | 3.48 | 3.56 | 3.90 | 3.61 | 3.54 | 4.66 | 4.63 | 3.52 | 3.36 | 3.70 | 3.63 | 3.43 |
| Mal | 5.91 | 5.59 | 5.14 | 4.69 | 4.76 | 5.92 | 5.20 | 5.43 | 5.50 | 5.04 | 5.48 | 5.75 | 5.79 | 5.80 | 5.88 | 6.16 |
| MI1 | 47.37 | 30.30 | 44.18 | 28.31 | 39.75 | 29.41 | 44.96 | 42.27 | 30.82 | 30.75 | 37.82 | 44.07 | 43.69 | 30.26 | 31.72 | 32.47 |
| MI2 | 67.63 | 85.39 | 61.02 | 69.77 | 65.38 | 85.29 | 64.74 | 64.39 | 87.87 | 65.38 | 55.99 | 58.98 | 59.19 | 79.47 | 79.88 | 86.65 |
| NAA1 | 18.48 | 18.23 | 18.43 | 18.68 | 20.54 | 19.93 | 18.75 | 18.40 | 20.39 | 16.46 | 16.26 | 19.89 | 18.18 | 18.83 | 18.37 | 20.61 |
| NAA2 | 225.12 | 196.14 | 221.75 | 176.65 | 223.88 | 207.61 | 224.51 | 215.72 | 215.07 | 122.47 | 174.67 | 241.87 | 218.81 | 184.94 | 176.09 | 232.60 |
| NAA3 | 28.02 | 21.06 | 26.38 | 22.61 | 28.76 | 22.89 | 27.67 | 26.69 | 23.04 | 23.20 | 22.40 | 28.23 | 26.54 | 23.42 | 24.14 | 20.64 |
| NAA4 | 28.28 | 29.21 | 27.35 | 29.27 | 31.76 | 32.01 | 28.64 | 28.41 | 31.73 | 25.74 | 22.66 | 29.00 | 26.61 | 30.16 | 29.39 | 30.47 |
| NAD1 | 0.97 | 0.99 | 1.08 | 1.00 | 0.93 | 1.25 | 1.23 | 1.08 | 1.33 | 0.95 | 0.70 | 1.14 | 1.21 | 0.96 | 1.30 | 1.21 |
| NAD2 | 0.56 | 0.65 | 0.65 | 0.59 | 0.60 | 0.73 | 0.71 | 0.58 | 0.82 | 0.60 | 0.48 | 0.67 | 0.75 | 0.61 | 0.82 | 0.65 |
| NAD3 | 0.64 | 0.56 | 0.69 | 0.67 | 0.60 | 0.77 | 0.73 | 0.64 | 0.75 | 0.57 | 0.53 | 0.67 | 0.73 | 0.57 | 0.80 | 0.70 |
| NADP1 | 0.14 | 0.10 | 0.19 | 0.11 | 0.11 | 0.10 | 0.10 | 0.17 | 0.14 | 0.05 | 0.10 | 0.11 | 0.16 | 0.12 | 0.13 | 0.17 |
| NADP2 | 0.09 | 0.08 | 0.10 | 0.07 | 0.10 | 0.09 | 0.09 | 0.10 | 0.12 | 0.03 | 0.05 | 0.06 | 0.08 | 0.07 | 0.13 | 0.10 |
| NADP3 | 0.07 | 0.04 | 0.04 | 0.12 | 0.06 | 0.05 | 0.05 | 0.01 | 0.08 | 0.02 | 0.05 | 0.06 | 0.07 | 0.05 | 0.03 | 0.08 |
| Nic1 | 0.48 | 0.53 | 0.50 | 0.39 | 0.58 | 0.38 | 0.43 | 0.41 | 0.44 | 0.33 | 0.54 | 0.39 | 0.43 | 0.56 | 0.44 | 0.62 |
| Nic2 | 0.38 | 0.46 | 0.36 | 0.33 | 0.49 | 0.39 | 0.31 | 0.31 | 0.32 | 0.24 | 0.43 | 0.33 | 0.35 | 0.48 | 0.34 | 0.46 |
| Nic3 | 0.39 | 0.35 | 0.32 | 0.30 | 0.43 | 0.28 | 0.30 | 0.34 | 0.31 | 0.19 | 0.37 | 0.29 | 0.31 | 0.35 | 0.28 | 0.40 |
| Pcho | 47.19 | 45.04 | 45.16 | 40.49 | 50.96 | 41.89 | 47.53 | 44.77 | 46.58 | 38.53 | 36.02 | 44.02 | 46.79 | 49.17 | 45.62 | 41.12 |
| PEA | 8.37 | 11.35 | 8.80 | 11.36 | 9.99 | 12.15 | 8.37 | 8.47 | 11.71 | 9.98 | 7.56 | 8.90 | 8.79 | 12.52 | 11.30 | 11.52 |
| Phe1 | 0.49 | 0.41 | 0.48 | 0.49 | 0.43 | 0.40 | 0.50 | 0.47 | 0.42 | 0.33 | 0.51 | 0.62 | 0.42 | 0.38 | 0.43 | 0.45 |
| Phe2 | 0.34 | 0.32 | 0.22 | 0.34 | 0.33 | 0.32 | 0.33 | 0.29 | 0.29 | 0.25 | 0.36 | 0.31 | 0.27 | 0.21 | 0.34 | 0.32 |
| Phe3 | 0.53 | 0.56 | 0.50 | 0.64 | 0.57 | 0.57 | 0.50 | 0.53 | 0.57 | 0.55 | 0.72 | 0.71 | 0.59 | 0.52 | 0.56 | 0.56 |
| Ser1 | 9.34 | 9.23 | 9.46 | 9.04 | 9.40 | 9.80 | 9.45 | 9.46 | 10.76 | 8.69 | 8.70 | 10.18 | 9.63 | 9.51 | 10.35 | 11.70 |
| Ser2 | 7.55 | 7.94 | 7.59 | 8.69 | 8.14 | 7.62 | 7.42 | 8.26 | 7.45 | 6.88 | 7.12 | 8.69 | 7.74 | 7.80 | 8.27 | 8.11 |
| Ser3 | 7.55 | 6.79 | 7.89 | 7.27 | 7.83 | 6.78 | 7.84 | 8.17 | 7.16 | 6.67 | 7.23 | 8.10 | 7.97 | 7.21 | 7.54 | 7.15 |
| Suc | 31.20 | 23.65 | 30.21 | 18.45 | 32.55 | 21.69 | 30.05 | 30.53 | 21.58 | 18.46 | 22.09 | 27.11 | 24.85 | 16.74 | 18.33 | 22.19 |
| Tau1 | 78.78 | 71.51 | 83.14 | 74.36 | 95.75 | 71.25 | 82.73 | 82.23 | 77.44 | 67.25 | 61.55 | 82.63 | 94.58 | 73.93 | 70.82 | 59.42 |
| Tau2 | 90.09 | 85.86 | 93.27 | 85.73 | 106.66 | 86.41 | 93.88 | 92.90 | 92.20 | 78.49 | 71.66 | 92.60 | 103.36 | 86.73 | 83.45 | 76.33 |
| Thr1 | 4.94 | 5.69 | 4.85 | 5.30 | 4.95 | 5.59 | 5.46 | 5.30 | 5.99 | 5.78 | 5.96 | 5.51 | 5.15 | 5.60 | 5.85 | 6.07 |
| Thr2 | 7.56 | 8.23 | 6.86 | 7.25 | 7.73 | 7.06 | 7.64 | 7.73 | 7.12 | 6.44 | 7.17 | 7.54 | 7.26 | 7.57 | 8.19 | 7.47 |
| Tyr1 | 0.84 | 0.64 | 0.63 | 0.69 | 0.64 | 0.52 | 0.70 | 0.68 | 0.57 | 0.60 | 0.59 | 0.68 | 0.73 | 0.47 | 0.69 | 0.52 |
| Tyr2 | 1.03 | 1.01 | 0.66 | 1.08 | 0.77 | 0.74 | 0.81 | 0.92 | 0.78 | 0.75 | 0.66 | 0.87 | 0.91 | 0.71 | 0.94 | 0.83 |
| UDPGa | 1.36 | 1.45 | 1.65 | 1.11 | 1.63 | 1.52 | 1.77 | 1.53 | 1.78 | 1.22 | 1.48 | 2.03 | 1.54 | 1.77 | 1.61 | 1.75 |
| undermine | 1.65 | 2.54 | 2.02 | 2.13 | 1.94 | 3.48 | 2.56 | 1.81 | 3.82 | 1.97 | 2.33 | 2.39 | 1.97 | 3.35 | 2.70 | 3.87 |
| Ura1 | 0.21 | 0.16 | 0.12 | 0.25 | 0.15 | 0.09 | 0.11 | 0.18 | 0.17 | 0.14 | 0.10 | 0.11 | 0.15 | 0.14 | 0.17 | 0.22 |
| Ura2 | 0.18 | 0.09 | 0.06 | 0.07 | 0.13 | 0.10 | 0.09 | 0.13 | 0.06 | 0.10 | 0.00 | 0.09 | 0.03 | 0.00 | 0.08 | 0.02 |
| Uri | 0.58 | 0.40 | 0.39 | 0.33 | 0.44 | 0.25 | 0.42 | 0.45 | 0.24 | 0.47 | 0.29 | 0.34 | 0.47 | 0.21 | 0.36 | 0.17 |
| Val1 | 3.91 | 3.60 | 3.46 | 3.80 | 3.98 | 3.66 | 3.67 | 3.86 | 3.67 | 4.88 | 4.89 | 3.34 | 3.46 | 3.69 | 3.58 | 3.59 |
| Val2 | 4.22 | 3.78 | 3.72 | 3.79 | 4.16 | 3.80 | 3.90 | 4.09 | 3.72 | 5.11 | 5.23 | 3.52 | 3.78 | 3.91 | 3.64 | 3.62 |

* There are two or more peaks of some metabolites in the NMR spectra, and then those peaks were named as the abbreviate name added with a number.
