# Supplementary material for: Oxidative stress, dysfunctional energy metabolism, and destabilizing neurotransmitters altered the cerebral metabolic profile in a rat model of simulated heliox saturation diving to 4.0 MPa
Source: PLoS One. 2023 Mar 14;18(3):e0282700. doi: 10.1371/journal.pone.0282700 (PMC10013885; doi:10.1371/journal.pone.0282700)
Supplement: S6 Table — (DOCX) [file pone.0282700.s007.docx]

**S6 Table. The peak height raw data of striatum samples.**

| Metabolites | striatum | | | | | | | | | | | | | | | |
| --- | --- | --- | --- | --- | --- | --- | --- | --- | --- | --- | --- | --- | --- | --- | --- | --- |
|  | CON1 | CON2 | CON3 | CON4 | CON5 | CON6 | CON7 | CON8 | HSD1 | HSD2 | HSD3 | HSD4 | HSD5 | HSD6 | HSD7 | HSD8 |
| 2HIB | 9.63 | 10.71 | 9.39 | 10.33 | 8.95 | 11.55 | 7.24 | 11.26 | 9.73 | 10.23 | 14.45 | 9.84 | 9.65 | 9.44 | 9.25 | 13.35 |
| Ala | 20.23 | 18.91 | 20.30 | 15.24 | 13.83 | 20.54 | 17.09 | 17.69 | 18.22 | 18.32 | 15.36 | 14.43 | 17.54 | 19.35 | 14.35 | 13.71 |
| AMP1* | 11.25 | 16.74 | 15.97 | 11.35 | 13.38 | 16.70 | 12.49 | 15.18 | 13.96 | 14.37 | 8.47 | 14.94 | 11.25 | 14.82 | 15.69 | 11.39 |
| AMP2* | 10.76 | 16.71 | 16.22 | 8.32 | 6.67 | 18.34 | 15.37 | 14.41 | 14.33 | 14.09 | 11.42 | 9.99 | 13.34 | 15.53 | 15.61 | 10.04 |
| AMP3 | 5.31 | 8.93 | 8.33 | 7.16 | 8.09 | 9.69 | 8.87 | 6.50 | 8.24 | 7.81 | 7.32 | 9.24 | 9.50 | 8.74 | 9.29 | 7.76 |
| AMP4 | 5.07 | 7.13 | 6.78 | 4.36 | 4.56 | 7.53 | 5.90 | 6.29 | 6.31 | 6.32 | 5.50 | 4.83 | 5.62 | 6.41 | 5.99 | 4.60 |
| AMP5 | 5.07 | 7.13 | 6.78 | 4.36 | 4.56 | 7.53 | 5.90 | 6.30 | 6.32 | 6.32 | 5.50 | 4.83 | 5.62 | 6.42 | 4.71 | 4.60 |
| AMP6 | 7.13 | 8.26 | 7.83 | 6.55 | 7.36 | 8.77 | 7.50 | 7.54 | 7.62 | 7.46 | 7.21 | 7.68 | 7.61 | 7.84 | 7.67 | 7.07 |
| Asc | 10.05 | 10.98 | 10.99 | 10.89 | 11.09 | 12.90 | 14.40 | 8.22 | 11.46 | 14.00 | 7.59 | 11.44 | 13.33 | 13.58 | 13.80 | 8.29 |
| Asn | 1.31 | 1.20 | 0.98 | 1.44 | 1.72 | 1.31 | 1.74 | 1.12 | 1.62 | 1.41 | 1.48 | 1.73 | 1.52 | 1.53 | 1.47 | 1.39 |
| Asp1 | 12.35 | 10.70 | 10.75 | 11.93 | 10.71 | 11.36 | 10.90 | 12.29 | 10.70 | 10.98 | 8.74 | 11.89 | 10.00 | 10.65 | 11.07 | 10.18 |
| Asp2 | 7.78 | 7.59 | 6.76 | 7.89 | 6.68 | 7.74 | 8.05 | 8.25 | 7.16 | 8.17 | 5.68 | 8.04 | 6.86 | 7.71 | 5.38 | 6.52 |
| Asp3 | 7.70 | 7.60 | 6.50 | 7.99 | 7.79 | 7.37 | 8.22 | 7.61 | 7.40 | 8.51 | 5.75 | 8.81 | 7.08 | 8.03 | 6.58 | 6.96 |
| ATP | 3.66 | 1.02 | 2.27 | 3.48 | 1.38 | 1.96 | 3.22 | 1.69 | 1.50 | 0.82 | 1.28 | 1.50 | 2.22 | 1.50 | 1.36 | 1.46 |
| Car | 2.73 | 2.65 | 2.83 | 2.62 | 3.01 | 3.33 | 3.19 | 3.30 | 3.16 | 2.07 | 2.81 | 2.58 | 3.01 | 2.41 | 2.74 | 2.37 |
| Cho | 34.22 | 20.84 | 25.19 | 24.59 | 27.08 | 21.86 | 22.13 | 30.92 | 18.74 | 10.70 | 13.54 | 20.30 | 17.26 | 15.01 | 19.91 | 14.24 |
| Cre | 195.51 | 189.86 | 198.67 | 179.73 | 147.64 | 210.73 | 199.48 | 168.73 | 183.22 | 183.35 | 137.55 | 157.79 | 174.42 | 194.62 | 140.08 | 130.98 |
| Cyt | 0.33 | 0.32 | 0.37 | 0.40 | 0.36 | 0.32 | 0.32 | 0.40 | 0.21 | 0.22 | 0.25 | 0.15 | 0.23 | 0.18 | 0.42 | 0.06 |
| DMA | 11.67 | 11.83 | 12.79 | 15.73 | 4.76 | 47.84 | 9.65 | 18.63 | 7.89 | 6.69 | 37.96 | 4.68 | 4.92 | 4.61 | 11.82 | 18.00 |
| FMA | 0.34 | 0.35 | 0.56 | 0.38 | 0.17 | 0.95 | 0.27 | 0.22 | 0.47 | 0.48 | 0.41 | 0.35 | 0.34 | 0.37 | 0.52 | 0.34 |
| For | 4.40 | 6.00 | 7.12 | 5.47 | 1.11 | 7.66 | 3.75 | 7.80 | 2.95 | 2.96 | 8.58 | 3.02 | 3.26 | 3.00 | 2.97 | 2.71 |
| GABA1 | 24.68 | 19.40 | 23.71 | 25.57 | 32.55 | 16.33 | 26.67 | 24.93 | 19.25 | 18.34 | 16.83 | 23.89 | 22.17 | 16.52 | 25.85 | 20.52 |
| GABA2 | 19.68 | 15.72 | 18.94 | 17.67 | 20.78 | 14.29 | 21.57 | 16.15 | 18.91 | 15.35 | 12.78 | 15.70 | 16.25 | 15.46 | 16.21 | 13.88 |
| GABA3 | 21.84 | 16.93 | 21.02 | 18.20 | 21.03 | 15.68 | 23.14 | 18.83 | 20.21 | 16.92 | 12.88 | 15.84 | 16.87 | 16.39 | 15.22 | 13.24 |
| Gln1 | 28.95 | 27.09 | 29.11 | 34.52 | 35.61 | 32.15 | 36.83 | 22.29 | 30.40 | 26.19 | 29.56 | 36.93 | 35.25 | 31.41 | 32.58 | 31.92 |
| Gln2 | 27.78 | 26.62 | 25.25 | 23.70 | 23.01 | 29.91 | 28.42 | 24.33 | 26.78 | 21.15 | 18.89 | 24.30 | 23.44 | 26.72 | 23.65 | 23.04 |
| Glu1 | 31.77 | 31.52 | 33.46 | 24.16 | 27.70 | 35.82 | 25.61 | 33.70 | 27.97 | 30.66 | 21.74 | 28.38 | 27.72 | 27.21 | 32.94 | 24.27 |
| Glu2 | 42.96 | 43.22 | 43.32 | 33.18 | 33.40 | 48.26 | 39.29 | 41.98 | 39.87 | 41.56 | 27.43 | 35.41 | 37.55 | 40.79 | 38.41 | 33.24 |
| Glu3 | 43.25 | 43.48 | 43.61 | 33.43 | 33.61 | 48.59 | 39.60 | 42.23 | 40.13 | 41.72 | 27.57 | 35.65 | 37.82 | 41.05 | 38.68 | 33.42 |
| Gly | 24.81 | 26.40 | 25.47 | 20.13 | 22.11 | 26.40 | 21.02 | 19.33 | 17.24 | 26.90 | 20.20 | 19.21 | 20.49 | 20.92 | 18.24 | 16.24 |
| GPC | 31.59 | 45.46 | 44.03 | 43.46 | 44.27 | 51.53 | 52.45 | 37.85 | 60.16 | 32.41 | 29.97 | 44.45 | 43.67 | 41.97 | 54.33 | 42.79 |
| GSH1 | 0.98 | 1.05 | 1.00 | 1.20 | 1.01 | 1.20 | 1.68 | 1.27 | 1.37 | 1.21 | 1.59 | 1.26 | 1.01 | 1.25 | 2.44 | 1.08 |
| GSH2 | 4.53 | 4.80 | 4.77 | 5.63 | 5.84 | 5.18 | 5.53 | 4.43 | 5.49 | 4.31 | 4.67 | 5.93 | 5.73 | 5.09 | 4.34 | 4.95 |
| GSH3 | 4.56 | 4.55 | 4.96 | 4.19 | 3.94 | 4.94 | 5.59 | 4.22 | 4.73 | 4.34 | 4.10 | 4.13 | 4.18 | 4.56 | 5.55 | 3.82 |
| GSH4 | 1.24 | 1.19 | 1.17 | 1.08 | 1.38 | 1.36 | 1.98 | 1.24 | 1.41 | 1.28 | 1.43 | 1.27 | 1.22 | 1.36 | 3.44 | 1.30 |
| Ile | 3.90 | 4.47 | 3.30 | 3.86 | 3.96 | 3.12 | 3.83 | 3.78 | 4.47 | 4.23 | 4.29 | 4.25 | 4.41 | 4.10 | 3.62 | 4.30 |
| IMP1 | 1.22 | 2.23 | 1.91 | 2.17 | 1.87 | 2.63 | 2.06 | 1.59 | 1.86 | 0.86 | 1.22 | 2.41 | 1.82 | 2.37 | 2.41 | 1.84 |
| IMP2 | 3.38 | 3.11 | 3.65 | 3.85 | 4.07 | 2.94 | 3.20 | 3.60 | 2.44 | 1.99 | 1.84 | 4.04 | 3.39 | 2.16 | 4.04 | 2.94 |
| Ino | 1.64 | 0.35 | 1.01 | 1.13 | 1.24 | 0.86 | 0.84 | 1.06 | 1.04 | 0.89 | 0.32 | 1.02 | 0.48 | 0.67 | 0.66 | 0.44 |
| Lac1 | 36.47 | 35.38 | 36.82 | 37.08 | 34.92 | 39.95 | 40.07 | 31.47 | 36.26 | 45.12 | 28.05 | 39.97 | 36.11 | 42.47 | 34.45 | 31.45 |
| Lac2 | 155.28 | 151.85 | 155.33 | 140.65 | 128.86 | 164.36 | 154.61 | 141.35 | 149.19 | 198.68 | 118.64 | 140.94 | 141.23 | 173.73 | 96.54 | 102.11 |
| Leu | 6.90 | 7.72 | 6.45 | 7.29 | 7.50 | 6.15 | 7.16 | 6.48 | 7.80 | 7.52 | 7.39 | 7.70 | 7.78 | 7.00 | 7.71 | 7.58 |
| Lys | 4.51 | 5.02 | 4.46 | 5.00 | 4.95 | 4.62 | 5.09 | 4.64 | 4.92 | 4.65 | 5.53 | 4.94 | 5.01 | 4.74 | 5.07 | 5.29 |
| Mal | 5.21 | 5.17 | 4.77 | 4.40 | 5.17 | 5.48 | 4.25 | 6.15 | 4.44 | 5.48 | 4.26 | 5.29 | 4.16 | 4.52 | 4.46 | 4.28 |
| MI1 | 29.80 | 27.23 | 28.98 | 21.47 | 22.84 | 29.96 | 24.10 | 29.02 | 27.63 | 37.59 | 20.98 | 18.58 | 19.61 | 27.29 | 21.58 | 17.55 |
| MI2 | 40.76 | 36.26 | 38.42 | 54.15 | 57.46 | 42.50 | 50.59 | 29.96 | 46.81 | 55.68 | 33.45 | 51.98 | 46.81 | 48.94 | 50.88 | 42.50 |
| NAA1 | 15.32 | 15.43 | 15.65 | 15.75 | 14.72 | 17.01 | 17.16 | 16.11 | 15.24 | 16.21 | 12.33 | 15.87 | 15.42 | 16.97 | 15.85 | 13.38 |
| NAA2 | 172.14 | 170.08 | 174.19 | 150.82 | 155.39 | 187.56 | 131.50 | 183.41 | 142.71 | 172.53 | 87.52 | 160.51 | 141.60 | 154.26 | 168.17 | 129.27 |
| NAA3 | 21.40 | 20.56 | 20.06 | 18.06 | 14.99 | 22.51 | 21.62 | 20.37 | 20.02 | 22.31 | 13.74 | 17.09 | 17.79 | 22.45 | 12.38 | 13.30 |
| NAA4 | 22.27 | 21.25 | 21.81 | 24.79 | 23.59 | 24.16 | 26.59 | 17.66 | 22.74 | 22.61 | 18.04 | 24.17 | 24.44 | 25.69 | 21.66 | 19.88 |
| NAD1 | 1.20 | 1.13 | 1.20 | 1.22 | 1.21 | 1.39 | 1.24 | 1.22 | 0.92 | 0.69 | 0.74 | 1.05 | 1.29 | 1.10 | 1.52 | 1.11 |
| NAD2 | 0.67 | 0.73 | 0.71 | 0.82 | 0.62 | 0.98 | 0.76 | 0.71 | 0.56 | 0.47 | 0.40 | 0.67 | 0.82 | 0.65 | 0.91 | 0.65 |
| NAD3 | 0.77 | 0.85 | 0.66 | 0.70 | 0.64 | 0.81 | 0.81 | 0.87 | 0.63 | 0.53 | 0.49 | 0.59 | 0.70 | 0.70 | 0.85 | 0.63 |
| NADP1 | 0.18 | 0.24 | 0.16 | 0.32 | 0.17 | 0.18 | 0.14 | 0.20 | 0.09 | 0.09 | 0.18 | 0.14 | 0.16 | 0.13 | 0.20 | 0.13 |
| NADP2 | 0.21 | 0.29 | 0.29 | 0.29 | 0.12 | 0.26 | 0.11 | 0.26 | 0.08 | 0.04 | 0.13 | 0.18 | 0.10 | 0.12 | 0.30 | 0.08 |
| NADP3 | 0.12 | 0.17 | 0.31 | 0.30 | 0.15 | 0.33 | 0.14 | 0.15 | 0.10 | 0.05 | 0.16 | 0.15 | 0.10 | 0.09 | 0.34 | 0.09 |
| Nic1 | 0.71 | 0.83 | 0.72 | 0.94 | 0.55 | 0.87 | 0.55 | 0.62 | 0.75 | 0.54 | 0.67 | 0.84 | 0.51 | 0.54 | 0.74 | 0.56 |
| Nic2 | 0.55 | 0.71 | 0.58 | 0.75 | 0.41 | 0.71 | 0.44 | 0.59 | 0.59 | 0.42 | 0.62 | 0.61 | 0.50 | 0.44 | 0.64 | 0.45 |
| Nic3 | 0.47 | 0.50 | 0.54 | 0.54 | 0.36 | 0.71 | 0.42 | 0.41 | 0.45 | 0.38 | 0.49 | 0.32 | 0.33 | 0.36 | 0.64 | 0.29 |
| Pcho | 66.56 | 65.08 | 69.52 | 73.38 | 62.04 | 75.19 | 72.29 | 54.06 | 65.77 | 35.79 | 53.90 | 65.99 | 66.79 | 60.94 | 67.56 | 53.06 |
| PEA | 12.46 | 12.59 | 12.94 | 15.48 | 15.81 | 14.42 | 16.12 | 10.16 | 13.90 | 7.54 | 12.32 | 15.82 | 16.32 | 12.37 | 16.97 | 13.18 |
| Phe1 | 0.50 | 0.61 | 0.62 | 0.36 | 0.45 | 0.66 | 0.47 | 0.40 | 0.41 | 0.51 | 0.36 | 0.50 | 0.44 | 0.56 | 0.57 | 0.38 |
| Phe2 | 0.36 | 0.42 | 0.49 | 0.28 | 0.40 | 0.63 | 0.46 | 0.53 | 0.35 | 0.36 | 0.48 | 0.47 | 0.37 | 0.45 | 0.56 | 0.31 |
| Phe3 | 1.08 | 1.40 | 1.88 | 1.51 | 1.08 | 1.22 | 0.91 | 1.06 | 0.81 | 0.73 | 1.41 | 0.99 | 0.88 | 0.76 | 1.54 | 0.95 |
| Ser1 | 8.98 | 8.99 | 8.97 | 8.96 | 8.85 | 9.39 | 9.43 | 8.48 | 9.19 | 8.71 | 8.20 | 9.50 | 9.01 | 9.96 | 8.91 | 9.01 |
| Ser2 | 7.36 | 6.74 | 6.75 | 7.47 | 6.75 | 7.33 | 6.79 | 6.97 | 6.80 | 7.14 | 6.92 | 7.39 | 6.94 | 7.48 | 5.96 | 7.01 |
| Ser3 | 7.42 | 7.02 | 7.25 | 6.28 | 6.02 | 7.10 | 6.42 | 7.01 | 6.95 | 7.25 | 6.55 | 6.04 | 6.40 | 7.30 | 4.94 | 5.93 |
| Suc | 30.24 | 23.67 | 32.16 | 16.72 | 24.99 | 28.59 | 21.02 | 26.41 | 26.02 | 21.96 | 13.52 | 20.42 | 20.54 | 22.05 | 26.29 | 18.87 |
| Tau1 | 107.54 | 95.57 | 111.10 | 100.36 | 71.86 | 108.87 | 109.96 | 92.29 | 96.67 | 61.12 | 71.79 | 83.05 | 94.77 | 91.61 | 68.53 | 59.83 |
| Tau2 | 111.81 | 98.87 | 114.76 | 104.77 | 80.67 | 114.08 | 113.81 | 94.43 | 102.93 | 71.16 | 84.00 | 88.07 | 97.75 | 98.60 | 79.76 | 70.88 |
| Thr1 | 6.19 | 6.26 | 6.30 | 6.67 | 6.31 | 6.41 | 6.07 | 6.43 | 5.65 | 5.99 | 6.73 | 6.79 | 6.08 | 6.43 | 6.50 | 6.87 |
| Thr2 | 8.83 | 8.07 | 7.57 | 8.24 | 8.48 | 8.52 | 8.68 | 6.28 | 7.59 | 7.18 | 6.98 | 8.72 | 8.01 | 8.23 | 7.62 | 7.79 |
| Tyr1 | 0.87 | 0.65 | 0.69 | 0.40 | 0.62 | 0.76 | 1.07 | 0.85 | 0.65 | 0.60 | 0.35 | 0.50 | 0.45 | 0.70 | 0.67 | 0.44 |
| Tyr2 | 1.14 | 1.07 | 1.07 | 0.86 | 0.98 | 0.92 | 1.30 | 0.99 | 0.73 | 0.67 | 0.86 | 0.94 | 0.74 | 0.86 | 0.93 | 0.73 |
| UDPGa | 1.01 | 1.69 | 1.42 | 0.91 | 1.09 | 1.83 | 1.53 | 1.38 | 1.52 | 1.49 | 1.27 | 1.39 | 1.42 | 1.66 | 1.33 | 1.20 |
| undermine | 2.11 | 2.22 | 1.76 | 2.75 | 2.82 | 2.63 | 2.22 | 1.46 | 2.12 | 2.31 | 2.08 | 3.18 | 2.35 | 2.48 | 2.99 | 2.45 |
| Ura1 | 0.29 | 0.33 | 0.33 | 0.26 | 0.19 | 0.46 | 0.28 | 0.39 | 0.14 | 0.11 | 0.29 | 0.23 | 0.15 | 0.13 | 0.46 | 0.25 |
| Ura2 | 0.01 | 0.00 | 0.06 | 0.00 | 0.07 | 0.00 | 0.08 | 0.01 | 0.12 | 0.00 | 0.18 | 0.00 | 0.00 | 0.02 | 0.11 | 0.00 |
| Uri | 0.49 | 0.00 | 0.15 | 0.24 | 0.23 | 0.22 | 0.37 | 0.18 | 0.24 | 0.31 | 0.15 | 0.15 | 0.25 | 0.22 | 0.06 | 0.12 |
| Val1 | 4.72 | 5.30 | 4.21 | 4.87 | 4.90 | 4.06 | 4.62 | 4.38 | 5.07 | 4.83 | 5.73 | 4.93 | 5.27 | 4.86 | 4.66 | 5.02 |
| Val2 | 5.03 | 5.66 | 4.44 | 5.00 | 5.14 | 4.24 | 4.91 | 4.59 | 5.41 | 5.18 | 5.27 | 5.24 | 5.53 | 5.08 | 4.82 | 5.38 |

* There are two or more peaks of some metabolites in the NMR spectra, and then those peaks were named as the abbreviate name added with a number.
