# Supplementary material for: Oxidative stress, dysfunctional energy metabolism, and destabilizing neurotransmitters altered the cerebral metabolic profile in a rat model of simulated heliox saturation diving to 4.0 MPa
Source: PLoS One. 2023 Mar 14;18(3):e0282700. doi: 10.1371/journal.pone.0282700 (PMC10013885; doi:10.1371/journal.pone.0282700)
Supplement: Attachment — Submitted filename: rebuttal lette20230104.docx [file pone.0282700.s008.docx]

**rebuttal letter**

Dear Reviewers and editors,

Thank you for giving us a chance to submit a revised version of the manuscript.

Journal Requirements:

When submitting your revision, we need you to address these additional requirements.

1. Please ensure that your manuscript meets PLOS ONE's style requirements, including those for file naming. The PLOS ONE style templates can be found at

https://journals.plos.org/plosone/s/file?id=wjVg/PLOSOne_formatting_sample_main_body.pdf and

<https://journals.plos.org/plosone/s/file?id=ba62/PLOSOne_formatting_sample_title_authors_affiliations.pdf>

Answer: We appreciate this comment. Changes have been made according to the manuscript format.

2. In the Methods section of your revised manuscript, please include the full name of the institutional review board or ethics committee that approved the protocol, the approval or permit number that was issued, and the date that approval was granted.

Answer: We appreciate this comment. Changes have been made as follow: All experimental protocols were approved by the Animal Ethics Committee of Naval Medical Center of PLA, Naval Medical University (Approval no: SYXK(Shanghai)2017-0019, Approval year: 2020).

3. Thank you for stating the following financial disclosure:

“No”

At this time, please address the following queries:

a)        Please clarify the sources of funding (financial or material support) for your study. List the grants or organizations that supported your study, including funding received from your institution.

b)        State what role the funders took in the study. If the funders had no role in your study, please state: “The funders had no role in study design, data collection and analysis, decision to publish, or preparation of the manuscript.”

c)        If any authors received a salary from any of your funders, please state which authors and which funders.

d)        If you did not receive any funding for this study, please state: “The authors received no specific funding for this work.”235-247-341

Please include your amended statements within your cover letter; we will change the online submission form on your behalf.

Answer: We appreciate this comment. This work was financially supported by the military medical innovation special project (CWS11J028) and the Thirteenth Five-Year Plan" military key disciplines and professional construction projects (Grant No. 2020SZ19-4; 22020SZ20-3; 2020SZ23-1; 2020SZ25). The funders had no role in study design, data collection and analysis, decision to publish, or preparation of the manuscript. Not any authors received a salary from any of above-mentioned funders.

4. We note that you have indicated that data from this study are available upon request. PLOS only allows data to be available upon request if there are legal or ethical restrictions on sharing data publicly. For more information on unacceptable data access restrictions, please see http://journals.plos.org/plosone/s/data-availability#loc-unacceptable-data-access-restrictions.

In your revised cover letter, please address the following prompts:

a) If there are ethical or legal restrictions on sharing a de-identified data set, please explain them in detail (e.g., data contain potentially sensitive information, data are owned by a third-party organization, etc.) and who has imposed them (e.g., an ethics committee). Please also provide contact information for a data access committee, ethics committee, or other institutional body to which data requests may be sent.

b) If there are no restrictions, please upload the minimal anonymized data set necessary to replicate your study findings as either Supporting Information files or to a stable, public repository and provide us with the relevant URLs, DOIs, or accession numbers. For a list of acceptable repositories, please see http://journals.plos.org/plosone/s/data-availability#loc-recommended-repositories.

We will update your Data Availability statement on your behalf to reflect the information you provide.

Answer: We appreciate this comment. Metabolomics data have been deposited to the EMBL-EBI MetaboLights database (DOI: 10.1093/nar/gkz1019, PMID:31691833) with the identifier MTBLS7185. The complete dataset can be accessed here https://www.ebi.ac.uk/metabolights/MTBLS7185.

5. PLOS requires an ORCID iD for the corresponding author in Editorial Manager on papers submitted after December 6th, 2016. Please ensure that you have an ORCID iD and that it is validated in Editorial Manager. To do this, go to ‘Update my Information’ (in the upper left-hand corner of the main menu), and click on the Fetch/Validate link next to the ORCID field. This will take you to the ORCID site and allow you to create a new iD or authenticate a pre-existing iD in Editorial Manager. Please see the following video for instructions on linking an ORCID iD to your Editorial Manager account: <https://www.youtube.com/watch?v=_xcclfuvtxQ>

Answer: We appreciate this comment. The ORCID iD of corresponding author (Ci Li) is 0000-0002-9572-1155.

6. We note that you have included the phrase “data not shown” in your manuscript. Unfortunately, this does not meet our data sharing requirements. PLOS does not permit references to inaccessible data. We require that authors provide all relevant data within the paper, Supporting Information files, or in an acceptable, public repository. Please add a citation to support this phrase or upload the data that corresponds with these findings to a stable repository (such as Figshare or Dryad) and provide and URLs, DOIs, or accession numbers that may be used to access these data. Or, if the data are not a core part of the research being presented in your study, we ask that you remove the phrase that refers to these data.

Answer: We appreciate this comment. We deleted these sentences of” In addition, we also investigated the serum metabolic profile changes in this research. Increased levels of isobutyrate and trimethylamine and decreased levels of lactate and pyruvate were present in the serum samples of the HSD group compared with those of the CON group (data not shown). Such a small number of differential metabolites in serum suggest that the brain tissue (34 discriminative metabolites) might be the target organ of hyperbaric exposure. Certainly, additional data are needed to draw more weighty conclusions.”

7. Your ethics statement should only appear in the Methods section of your manuscript. If your ethics statement is written in any section besides the Methods, please move it to the Methods section and delete it from any other section. Please ensure that your ethics statement is included in your manuscript, as the ethics statement entered into the online submission form will not be published alongside your manuscript.

Answer: We appreciate this comment. We move the “Ethics approval” to the methods.

8. Please include captions for your Supporting Information files at the end of your manuscript, and update any in-text citations to match accordingly. Please see our Supporting Information guidelines for more information: <http://journals.plos.org/plosone/s/supporting-information>.

Answer: We appreciate this comment. We added “Additional file: Additional file 1: Supplementary materials. “in the end of manuscript.

9. Please review your reference list to ensure that it is complete and correct. If you have cited papers that have been retracted, please include the rationale for doing so in the manuscript text, or remove these references and replace them with relevant current references. Any changes to the reference list should be mentioned in the rebuttal letter that accompanies your revised manuscript. If you need to cite a retracted article, indicate the article’s retracted status in the References list and also include a citation and full reference for the retraction notice.
Answer: We appreciate this comment. We reviewed the reference list. We didn’t cite any paper that have been retracted.

Reviewers' comments:

Reviewer's Responses to Questions

**Comments to the Author**

1. Is the manuscript technically sound, and do the data support the conclusions?

The manuscript must describe a technically sound piece of scientific research with data that supports the conclusions. Experiments must have been conducted rigorously, with appropriate controls, replication, and sample sizes. The conclusions must be drawn appropriately based on the data presented.

Reviewer #1: Partly

Reviewer #2: Yes

2. Has the statistical analysis been performed appropriately and rigorously?

Reviewer #1: Yes

Reviewer #2: Yes

3. Have the authors made all data underlying the findings in their manuscript fully available?

The [PLOS Data policy](http://www.plosone.org/static/policies.action#sharing) requires authors to make all data underlying the findings described in their manuscript fully available without restriction, with rare exception (please refer to the Data Availability Statement in the manuscript PDF file). The data should be provided as part of the manuscript or its supporting information, or deposited to a public repository. For example, in addition to summary statistics, the data points behind means, medians and variance measures should be available. If there are restrictions on publicly sharing data—e.g. participant privacy or use of data from a third party—those must be specified.

Reviewer #1: Yes

Reviewer #2: Yes

4. Is the manuscript presented in an intelligible fashion and written in standard English?

PLOS ONE does not copyedit accepted manuscripts, so the language in submitted articles must be clear, correct, and unambiguous. Any typographical or grammatical errors should be corrected at revision, so please note any specific errors here.

Reviewer #1: No

Reviewer #2: Yes

5. Review Comments to the Author

Please use the space provided to explain your answers to the questions above. You may also include additional comments for the author, including concerns about dual publication, research ethics, or publication ethics. (Please upload your review as an attachment if it exceeds 20,000 characters)

Reviewer #1: The findings are interesting; however, the conclusion of manuscript should be evaluated as the changes in the neurotransmitter and other prooxidant and antioxidant parameters shown were transient or irreversible in nature. Please elaborate?
Answer: We appreciate this comment. Whether the changes in the neurotransmitter and other prooxidant and antioxidant parameters shown were transient and irreversible in nature in the HSD group, we will elaborate in a future paper.
Is there any effect of pentobarbital sodium perse on the levels of neurotransmitter?
Answer: We appreciate this comment. We searched the literatures and found that Pentobarbital antagonizes the A1 adenosine receptor-mediated inhibition of hippocampal neurotransmitter release. Thus, there is some effect of pentobarbital sodium perse on the levels of neurotransmitter. But all the rats received pentobarbital sodium dose proportional to theirs body weight, the effect might also proportionate to body weight, the body weight between HSD and CON groups showed no statistically significant meaning. Thus，We can infer that the statistical pattern of neurotransmitter changes is not affected by pentobarbital sodium. Of course, we will confirm this statement in subsequent experimental studies where animals are not given sodium pentobarbital.

Please be consistent with the style of writing like “Five-hundred microliters” or 550 μL.
Answer: We appreciate this comment. “Five-hundred microliters” was changed as “an aliquot of 500 μL”.
Please abbreviate different labelling for the groups in the beginning “CONS”, “CONH”, “CONC”; “HSDS”, HSDC”, “HSDH”
Answer: We appreciate this comment. We changed the sentence” Cortex, hippocampus, and striatum tissues were dissected” as “Cortex, hippocampus, and striatum tissues were dissected, and abbreviated respectively as HSDC, HSDH, HSDS, CONC, CONH and CONS in two animal groups”.
Figure 3 legend labelling “cerebral” please correct
Answer: We appreciate this comment. we changed the cerebral as “cortex”.
Please mention the limit of detection of the ELISA kits used in the present study
Answer: We appreciate this comment. We listed the information of assay kits in the supply materials.

| Company | Product name | Limit of detection |
| --- | --- | --- |
| Abcam | ab83355 ATP Assay Kit | 1 µM |
| Abcam | ab235937 Cholinesterase Activity Assay Kit | 1 mU/ml |
| Abcam | ab102526 Lactate Dehydrogenase Assay Kit | 1 mU/ml |
| RD | Universal Dopamine ELISA Kit | 18.75 pg/mL |
| Abnova | KA3768 Epinephrine/Norepinephrine ELISA Kit | Adrenaline: 0.25 ng/mL |
| Abnova | KA3768 Epinephrine/Norepinephrine ELISA Kit | Noradrenaline: 0.1 ng/mL |
| BioSource | Rat 5-Hydroxytryptamine Assay Kit | 1.0 ng/mL |
| Santa Cruz | Rat Gamma-aminobutyric acid (GABA) Assay Kit | 0.1μmol/mL |
| cayman | Rat Super Oxidase Dimutase (SOD) ELISA Kit | 1.0 U/mL |
| cayman | Rat malondialchehyche (MDA) ELISA Kit | 0.1 nmol/mL |
| cayman | Rat Glutathione peroxidase (GSH-Px) ELISA Kit | 1.0 U/mL |

Table 1- Please correct “nmo” and “nmol”
Answer: We appreciate this comment. We corrected “nmo” and “nmol”.
Table 1 – For epinephrine value the standard deviation between CON and HSD is quite high to have a significant value? Please check
Answer: We appreciate this comment. A decimal point has been omitted and “11.42±119” of HSD was changed as “11.42±1.19”. we are very sorry for this mistake.
What does the dotted and straight lines indicate in figure 7? Please mention.
Answer: We appreciate this comment. The dotted lines in the figure 7 mean that two or more Intermediate entities or steps were needed in the transformation process of the first metabolites to the second metabolites. The straight lines mean that metabolites A can converted to metabolites B in one step reaction.
Figure 6C – Table is not spaced properly to analyze the data.
Answer: We appreciate this comment and added lines inside the table, thus data can be analyzed properly.
Figure 6C – Please mention the use of color and what it denotes?

Answer: We appreciate this comment, the shades of color in the circles in the Figure 6C stands for the value of -log10(p), the higher the redder, the lower the color become white from yellow.
There is no mention of metabolic enzymes, neurotransmitter levels and oxidative stress indicators in hippocampus and striatum. Please explain?

Answer: We appreciate this comment, for the volume and weight of hippocampus and striatum were so small, thus the separated tissues from rat’s brain were only for the detection of NMR-based metabolome.

Reviewer #2: The research article “Oxidative stress, dysfunctional energy metabolism, and destabilizing neurotransmitters altered cerebral metabolic profile in a rat model of a simulated heliox saturation diving to 4.0 Mpa” by Liu et al is very interesting and is expected to add significant new information in the research area of harmful effects of deep sea diving. However, I have some minor concerns mentioned below:
1. Could the metabolomic and biochemical alterations in rat brain induced by HSD be reversible? Has the author tried to assess these parameters after giving rats some recovery time?

Answer: We appreciate this comment. Whether the metabolomic and biochemical alterations in rat brain induced by HSD could be reversible, we will observe the changes of these parameters in the future by trying to give rats some recovery time.
2. Are the observed changes dependent on the time for which diving is performed?

Answer: We appreciate this comment. we will investigate whether the observation will change dependent on the diving time in the future.

6. PLOS authors have the option to publish the peer review history of their article ([what does this mean?](https://journals.plos.org/plosone/s/editorial-and-peer-review-process#loc-peer-review-history)). If published, this will include your full peer review and any attached files.


If you choose “no”, your identity will remain anonymous but your review may still be made public.


**Do you want your identity to be public for this peer review?** For information about this choice, including consent withdrawal, please see our [Privacy Policy](https://www.plos.org/privacy-policy).

Reviewer #1: No

Reviewer #2: No

[NOTE: If reviewer comments were submitted as an attachment file, they will be attached to this email and accessible via the submission site. Please log into your account, locate the manuscript record, and check for the action link "View Attachments". If this link does not appear, there are no attachment files.]

While revising your submission, please upload your figure files to the Preflight Analysis and Conversion Engine (PACE) digital diagnostic tool, <https://pacev2.apexcovantage.com/>. PACE helps ensure that figures meet PLOS requirements. To use PACE, you must first register as a user. Registration is free. Then, login and navigate to the UPLOAD tab, where you will find detailed instructions on how to use the tool. If you encounter any issues or have any questions when using PACE, please email PLOS at [figures@plos.org](mailto:figures@plos.org). Please note that Supporting Information files do not need this step.

*In compliance with data protection regulations, you may request that we remove your personal registration details at any time.* [*(Remove my information/details)*](https://www.editorialmanager.com/pone/login.asp?a=r)*. Please contact the publication office if you have any questions.*
